# Trajectories of Self-Injurious Thoughts and Behavior: Risk and Resiliency Among Cisgender and Gender Diverse Youth

**DOI:** 10.1016/j.jaacop.2025.10.006

**Published:** 2025-10-22

**Authors:** Amanda J. Thompson, Avery N. Abel, Rui Huang, Katherine Sarkisian, Mindy Westlund Schreiner, Franky Rife, Donna A. Ruch, Jeffrey A. Bridge

**Affiliations:** a The Center for Suicide Prevention and Research, Abigail Wexner Research Institute at Nationwide Children’s Hospital, Columbus, Ohio; b The Ohio State University College of Medicine, Columbus, Ohio

**Keywords:** self-injurious thoughts and behavior, transgender, gender diverse youth, suicide attempt

## Abstract

**Objective:**

Transgender and gender diverse (TGD) youth are at high risk for self-injurious thoughts and behaviors (SITB) including suicidal ideation, nonsuicidal self-injury (NSSI), and suicide attempt. We compared total SITB endorsements during a 4-year period among 3 groups: TGD youth with high gender-related social stress (TGD+High-Stress); TGD youth with low gender-related social stress (TGD+Low-Stress); and non-TGD youth. We further identified risk and resiliency correlates of 3 longitudinal SITB trajectories (NSSI, suicidal ideation, and suicide attempt), accounting for gender-related social stress and other known robust risk factors.

**Method:**

This study (N = 11,851) used longitudinal data for youth spanning ages 10 to 14 years from the Adolescent Brain Cognitive Development study (release 5.1), of whom 4% were TGD. Analyses of variance were used to compare mean SITB endorsements across groups. Three mixed-effects logistic regressions identified correlates of SITB trajectories during the study.

**Results:**

On average, TGD+High-Stress experienced more SITB events than TGD+Low-Stress and non-TGD youth, respectively. Longitudinal results found that TGD compared to non-TGD youth experienced higher NSSI and suicidal ideation risk regardless of gender-related social stress. TGD+High-Stress but not TGD+Low-Stress youth had greater suicide attempt risk than non-TGD youth. Higher psychopathology symptoms and family conflict were associated with higher NSSI and suicidal ideation risk. Only school involvement was protective against ideation and NSSI risk.

**Conclusion:**

TGD youth experience higher SITB risk, particularly when facing higher gender-related social stressors at home or school. We urgently need interventions supporting positive connections between TGD youth and their families and peers.

## Plain language summary

Using longitudinal data from the Adolescent Brain Cognitive Development℠ (ABCD) Study (N = 11,851), the authors compared total self-injurious thoughts and behaviors (SITB) among transgender and gender diverse (TGD) youth and non-TGD youth from ages 10 to 14 years. This study found that TGD youth experience higher risk for SITB than non-TGD youth. High gender-related social stress, more mental health symptoms, and family conflict were associated with higher risk of SITB, whereas school involvement was protective against SITB. This study highlights the need for developing interventions that support positive connections between TGD youth and their families and peers.

The American Academy of Pediatrics (AAP) defines transgender youth as including youth whose gender identity does not align with sex designated at birth (also referred to as gender incongruence) and can include transgender male, transgender female, nonbinary, gender fluid, gender queer, and other genders.^1^ Gender identity development is a typical process that unfolds through a series of complex interactions among a child’s environment, culture, and biology. Gender identity exploration and self-recognition of gender are normative processes within gender identity development that occur over time.^2^ Gender diverse youth, or gender non-conforming youth, are youth who express attitudes, display behaviors, or express beliefs that fall outside of cultural norms.^1^

Compared to cisgender youth (eg, youth whose gender identity aligns with sex designated at birth), transgender, and gender diverse youth experience a lifetime history of nonsuicidal self-injury (NSSI)^3^ and a lifetime history of suicidal ideation (SI)^4^ at prevalence rates that are triple the rates of those in cisgender youth.^4^ Transgender and gender diverse youth are also much more likely to experience a lifetime history of suicide attempt (SA) compared to cisgender youth.^5^^,^^6^ During 2013 to 2016, 59% of suicides in gender and sexual minoritized youth occurring between the ages of 12 and 17 years had stressors related to their gender identity or sexuality documented as circumstances contributing to their suicide.^7^ Suicidal ideation (SI), SA, and NSSI are strong risk factors for suicide,^8^^,^^9^ and thus are important targets for transgender and gender diverse (TGD) youth suicide prevention. Given that self-injurious thoughts and behavior (SITB)—including SI, SA, and NSSI—disproportionately affect TGD youth,^6^^,^^10^ research identifying risk and protective factors of SITB in TGD youth is urgently needed. At present, it is unclear what protects against SITB in TGD youth over and above the social stress that these youth face related to their gender identity in schools and home. Such research has a strong potential to inform tailored interventions and preventions for this population.

In 2024, nearly all surveyed TGD youth (94%) reported that recent US state and federal policy changes negatively affected their mental health.^11^ Still, 48 anti-transgender laws across multiple states were enacted from 2018 to 2022; implementation of these state-level policies preceded increased state-level past-year SA history rates among TGD youth.^12^ The minority stress model posits that gender identity does not singularly explain elevated SITB risk among TGD youth.^13^ Rather, elevated SITB risk among TGD youth is attributable to stressors such as discrimination, peer and family non-acceptance, rejection, internalized trans-negative gender norms, and concealing of gender identity.^13^, ^14^, ^15^ TGD youth, compared to cisgender youth, are at disproportionately elevated risk for SITB and SITB-associated mental health conditions, including internalizing disorders (eg, depression) and externalizing disorders (eg, disruptive disorders).^16^, ^17^, ^18^ However, research examining factors of risk and resiliency above and beyond gender-related stress in TGD youth is extremely limited.

In a study by Taliaferro *et al.*,^19^ economically disadvantaged high school–aged TGD youth experienced greater NSSI risk and co-occurring NSSI+SA risk compared to other TGD peers.^19^ Family conflict^20^, ^21^, ^22^ and lower feelings of school safety and connectedness are also associated with greater SITB risk in youth in general and in TGD youth in particular.^19^^,^^23^ Protective factors for TGD youth SITB remain understudied.^23^ Positive dimensions of school climate (eg, engagement, involvement, and safety) are generally protective against SITB and may be especially protective for TGD youth.^19^^,^^23^, ^24^, ^25^ Supportive families, particularly caregiver and sibling acceptance of gender identity and expression, are also protective against SITB in TGD youth.^19^^,^^21^^,^^23^ These positive connections with families and schools could potentially help protect against SA among TGD youth with NSSI.^19^

Despite higher SITB risk among TGD compared to cisgender youth,^3^^,^^5^ little is known about risk and resiliency factors of longitudinal SITB trajectories and the effect of gender-related social stress for TGD youth. Research that identifies early-emerging risk and protective factors could inform targeted preventions that would intervene earlier and shift youth away from SITB risk. Of the few existing longitudinal studies, TGD youth with prior SITB history were excluded despite being at high risk for suicide.^26^ Evaluating the role of gender-related social stress that TGD youth face is a critical next step that is currently lacking in existing longitudinal SITB studies.

The present study extends previous research by examining distal risk and resiliency correlates for NSSI, SI, and SA trajectories. We compared total numbers of SA, SI, and NSSI endorsements during the study, and hypothesized that TGD youth with higher levels of gender-related social stress would have more SITB experiences on average compared to TGD youth with lower levels of stress and non-TGD youth, respectively. The goal of the longitudinal analyses was to identify correlates of risk and resiliency accounting for gender-related social stress that TGD youth face compared to non-TGD youth. We hypothesized that higher internalizing problems, externalizing problems, and family conflict would be associated with riskier SITB trajectories in TGD compared to non-TGD youth regardless of gender-related social stress, whereas higher school involvement and caregiver acceptance would be associated with less risky SITB trajectories.

## Method

This study uses the first 5 annual assessments of the Adolescent Brain Cognitive Development^SM^ Study (ABCD Study) (5.1 release; doi.org/10.15154/z563-zd24), including 11,868 parent–child dyads (primary caregivers). The ABCD Study® is the largest prospective longitudinal study of youth in the United States, spanning multiple cities and beginning in 2017. The study began when children were ∼9 years old; the present study ended when participants were ∼15 years of age. Additional study details are described elsewhere.^27^

### Measures

#### Baseline Covariates

Covariates included children’s sex designated at birth, baseline age, and material and economic hardship—a measure more sensitive to socioeconomic differences across demographic groups compared to income.^28^ These covariates were included in all analyses, given established associations with risk for SITB. For example, male sex is associated with SITB at younger ages, whereas female sex is associated with more repeated SITB experiences and later onset.^29^^,^^30^ Because SA and NSSI were rare within certain racial groups (<10), and because <10 youth who were TGD was observed within certain racial groups, we collapsed racial groups into categories with cell sizes >10 in accordance with ABCD Study data use guidelines. Race and ethnicity were defined as non-Hispanic Black, Hispanic, non-Hispanic White, and Other (defined as non-Hispanic multiracial, American Indian/Alaskan Native, Asian American, Native Hawaiian, or other Pacific Islander). Still, longitudinal models would not converge with race included in the model (some racial groups had no SITB events). Prior research has examined SITB across race using ABCD Study data, and, given that our primary aim was unrelated to race, we excluded race from final analyses.^31^

#### Stressors That TGD Youth Experience

As part of the Schedule for Affective Disorders and Schizophrenia for School-Age Children—Present and Lifetime version (K-SADS-PL),^32^ youth were annually asked, “Are you transgender?” We excluded youth who never answered this question and youth whose only available data recorded that they did not understand the question (N = 11,581). We retrospectively assigned youth to groups. We defined the non-TGD group as youth who responded “No” to the question “Are you transgender?” at all participating assessments. We define transgender or gender diverse (TGD) youth as those who responded “Yes” or “Maybe” at least once, indicating some level of gender exploration, gender non-congruence, or transgender gender identity during the study.^33^ Participants responding with “Yes” or “Maybe” were asked, “Has this caused any problems for you with your family or with kids at school?” Youth could then report the level of problems that they experienced. These responses were recoded (0 = Not at all, 1 = Some, 2 = A lot). We defined 2 TGD groups as follows: (1) TGD+Low-Stress youth represent youth with lower levels or less persistent gender-related stress, and included youth who endorsed “Not at all” at all timepoints, or “Some” no more than once, and no endorsement of “A lot”; and (2) TGD+High-Stress represent youth with higher levels or enduring gender-related stress who endorsed “A lot” at least once, or “Some” at 2 or more timepoints. Importantly, this item examines external social stress that TGD youth experience during interactions with peers or family. This should not be interpreted as gender identity or gender identity exploration being problematic. Thus, we refer to these groups as having experienced gender-related social stress.

#### Baseline Risk and Protective Factors of SITB

We included primary caregivers’ reports of children’s broad internalizing and externalizing problems *T* scores using the Child Behavior Checklist (CBCL).^34^ We included child-reported parental acceptance (positive social interactions with primary caregiver) using the Parental Behavioral Inventory,^35^ family conflict using the Family Environment and Conflict Scale,^36^^,^^37^ and school involvement using the School Risk and Protective Factors scale.^38^ We controlled for whether youth started the study with a lifetime history of SITB.

#### SITB Trajectories

Caregivers completed the K-SADS-PL suicidality module biennially; children completed the suicidality module annually. Given that youth often do not disclose known history of self-harm at follow-up assessments and that self-harm is often under-reported,^39^^,^^40^ we defined youth as having NSSI, SI (passive or active), or SA (including aborted or interrupted attempts) if either youth or parent disclosed NSSI, SI, or SA. At baseline, many participants reported a lifetime history of NSSI (n = 597, 73%), SI (n = 1,257, 82%), and SA (n = 139, 85%) that was not current (within the past ≤2 weeks). Thus, baseline lifetime history for NSSI, SI, and SA were used as covariates in all 3 models. After baseline, each SITB event was assumed to have occurred since the last assessment; youth could have <5 positive endorsements for each outcome (current at baseline and at 1-year-, 2-year-, 3-year-, and 4-year-follow-up). NSSI, SI, and SA were treated as random effects in each of their respective models to examine the risk of experiencing SITB at least once during the study period. Additional measurement details including variables defining SITB are described in greater detail in the Supplemental Material, available online.

### Data Analysis

Sample descriptives and characteristics associated with missing data were analyzed using SPSS V26.^41^ Youth characteristics were significantly associated with missing data patterns (detailed results in Supplemental Material, available online). Multiple imputation of 5 datasets was used to address missing data on covariates and SITB (no data related to gender were imputed given that these data were necessary for study inclusion criteria), which can be appropriate for data that are unlikely to be missing completely at random.^42^^,^^43^ We fit 3 multilevel models: 2-level hierarchical models with repeated measures for each SITB outcome specified as an intercept and slope, using SAS (Enterprise Guide 8.5).^44^ A binomial distribution and logit link was specified for each of the 3 outcomes (NSSI, SI, and SA) measured across the 4 years of follow-up. Each model included a random intercept and a random slope for age by participant (years, rounded to the nearest tenth), thus accounting for individually varying observations of time between assessments, distinguishing between the fixed effect of age (the sample-average slope) and the random effect of age (the individual-specific deviations from that sample-average slope in risk over time).

Random effects (within-person differences) for each of the 3 outcomes (NSSI, SI, and SA) were modeled separately, allowing us to account for linear dependency of repeated measures when examining risk for positive endorsement of SITB with increasing age. The same series of fixed effects (between-person differences) were included in all 3 models and measured as before the 4-year study period. Results should be interpreted such that the intercept represents population average of outcome over time, and the regression coefficient represents slope over time.

Fixed effects for all longitudinal models included 2 dummy-coded variables comparing TGD+High-Stress and TGD+Low-Stress youth to the reference group of non-TGD youth, parental acceptance, family conflict, school involvement, internalizing problems, externalizing problems, material and economic deprivation, sex designated at birth, baseline age, and lifetime history of NSSI, SI, and SA. Given that we conducted a series of multiple tests in a large sample, we set a conservative alpha for interpreting significance (*p* ≤ .001).

## Results

### Sample Characteristics

The sample (N = 11,579) was 52% male and 47% female; 15% were Black non-Hispanic, 20% were Hispanic, 12% Other, 53% were White non-Hispanic, and less than 1% had missing data (Table 1 provides for additional participant information by TGD and non-TGD youth). Youth were 9.9 (SD = 0.62) years at baseline and 14.1 (SD = 0.68) years at the last assessment. There were 11,091 (95.8%) non-TGD youth, 379 (3.2%) TGD+Low-Stress youth, and 111 TGD+High-Stress youth (0.9%). The TGD+Low-Stress group had lower levels of gender-related stress and tended to affirm that they were TGD at younger ages, whereas the TGD+High-Stress group experienced higher gender-related stress and tended to affirm that they were TGD at older ages. Mean gender-related stress and percentage of youth positively affirming the transgender item within each time point are described further in the Supplemental Material (Tables S1 and S2, Figures S1 and S2, available online).

**Table 1:** Descriptive Statistics by Gender Identity and Stress Grouping

	Non-TGD	TGD+Low-Stress	TGD+High-Stress	Total sample
	(n = 11,091)	(n = 379)	(n = 111)	(N = 11,581)
Family conflict	2.04 (1.95)	2.08 (1.92)	2.33 (2.13)	2.04 (1.95)
Internalizing	48.32 (10.57)	50.50 (11.49)	51.65 (10.94)	48.43 (10.61)
Externalizing	45.62 (10.32)	46.99 (10.67)	47.46 (10.20)	45.68 (10.33)
Acceptance	2.78 (0.30)	2.76 (0.30)	2.61 (0.42)	2.78 (0.30)
Involvement	13.08 (2.35)	12.68 (2.54)	12.84 (2.48)	13.06 (2.36)
Deprivation	0.067 (0.159)	0.062 (0.150)	0.076 (0.149)	0.067 (0.158)
Age, y	9.93 (0.62)	9.80 (0.60)	9.95 (0.62)	9.93 (0.62)
Female sex, %	46.0	77.0	87.0	48.0
Black, %	15.0	6.0	12.0	15.0
Hispanic, %	20.0	19.0	14.0	20.0
White, %	52.0	59.0	57.0	53.0
Other race, %	13.0	16.0	17.0	13.0
Baseline lifetime SI, %	12.0	25.0	26.0	13.0
Baseline lifetime NSSI, %	7.0	13.0	16.0	7.0
Baseline lifetime SA, %	1.0	4.0	a	1.0

Note: TGD+Low-Stress and TGD+High-Stress are compared to reference group of non-TGD. Valid sample sizes range from approximately 11,057 to 11,090 for the non-TGD group, 378 to 379 for the TGD+Low-Stress group, and 111 consistently for the TGD+High-Stress group, depending on variable and missing data. Total sample sizes range from 11,547 to 11,580. Female sex = sex designated at birth as female compared to male; NSSI = nonsuicidal self-injury; SA = suicide attempt; SI = suicidal ideation; TGD = transgender and gender diverse; TGD+High-Stress = youth positively endorsing transgender item with high stress; TGD+Low-Stress = youth positively endorsing transgender item with low stress.

a Suppressed, n < 11.

### SITB Frequency Across Groups

There were significant differences in the mean number of positive endorsements during the study period between the 3 groups for the mean total number of positive NSSI, *F*_2,11579_ = 229.42, p < .001, η^2^ = 0.04, SI, *F*_2,11579_ = 120.54, p < .001, η^2^ = 0.04, and SA, *F*_2,11579_ = 231.07, η^2^ = 0.02, endorsements during the study period. Results from the analysis of variance (ANOVA) revealed the groups were significantly different on all outcomes. Post hoc comparisons found that TGD+High-Stress youth (mean_NSSI_ = 1.03, SD = 0.91, mean_SI_ = 1.18, SD = 0.87, mean_SA_ = 0.35, SD = 0.50) and TGD+Low-Stress youth (mean_NSSI_ = 0.58, SD = 0.72, mean_SI_ = 0.78, SD = 0.77, mean_SA_ = 0.17, SD = 0.38) had on average a greater number of positive NSSI, SI, and SA endorsements compared to non-TGD youth, respectively (mean_NSSI_ = 0.23, SD = 0.48, mean_SI_ = 0.33, SD = 0.56, mean_SA_ = 0.05, SD = 0.24).

### Longitudinal Trajectories of SITB

Table 2, Table 3, Table 4 provide model fit statistics, regression coefficients (log odds of endorsement risk), and model intercepts for each longitudinal SITB model described below.

**Table 2:** Risk for Nonsuicidal Self-Injury Over Time

	Regr. coeff.	SD	*t*	*p*	OR	95% CI
Intercept	–5.79	0.3	–16.24	<.001	0.003	0.002-0.006
Risk factors						
TGD+High-Stress	1.60	0.18	9.07	<.001	4.96	3.51-7.02
TGD+Low-Stress	0.83	0.11	7.44	<.001	2.30	1.85-2.87
Externalizing	0.01	0.00	4.00	<.001	1.01	1.01-1.02
Family conflict	0.05	0.01	3.71	.000	1.05	1.02-1.08
Internalizing	0.02	0.00	6.47	<.001	1.02	1.01-1.03
Protective factors						
Acceptance	–0.15	0.09	–1.67	.096	0.86	0.72-1.03
Involvement	–0.05	0.01	–4.87	<.001	0.95	0.93-0.97
Covariates						
Age	0.11	0.02	6.91	<.001	1.12	1.08-1.16
Deprivation	0.30	0.15	2.00	.045	1.35	1.01-1.82
Female sex	0.45	0.05	8.49	<.001	1.57	1.42-1.75
SI history	0.54	0.07	7.60	<.001	1.71	1.49-1.96
SA history	0.19	0.16	1.16	.245	1.21	0.88-1.67
NSSI history	0.69	0.09	8.13	<.001	2.00	1.69-2.36

Note: TGD+Low-Stress and TGD+High-Stress youth are compared to reference group of non-TGD youth. NSSI = nonsuicidal self-injury; OR = odds ratio; Regr. coeff. = regression coefficient; SA = suicide attempt; SI = suicidal ideation; TGD = transgender and gender diverse; TGD+High-Stress = youth positively endorsing transgender item with high stress; TGD+Low-Stress = youth positively endorsing transgender item with low stress.

**Table 3:** Risk for Suicidal Ideation Over Time

	SI (N = 11, 579)					
	Regr. coeff.	SD	*t*	*p*	OR	95% CI
Intercept	–6.02	0.61	–9.80	<.001	0.00	0.00-0.00
Risk factors						
TGD+High-Stress	1.45	0.16	9.35	<.001	4.26	3.14-5.78
TGD+Low-Stress	0.78	0.11	7.38	<.001	2.18	1.77-2.69
Externalizing	0.02	0.00	6.26	<.001	1.02	1.01-1.02
Family conflict	0.06	0.01	5.26	<.001	1.07	1.04-1.09
Internalizing	0.02	0.00	5.94	<.001	1.02	1.01-1.02
Protective factors						
Acceptance	–0.23	0.08	–2.90	.005	0.79	0.68-0.93
Involvement	–0.07	0.01	–6.80	<.001	0.94	0.92-0.95
Covariates						
Age	0.20	0.05	4.19	<.001	1.22	1.11-1.34
Deprivation	0.43	0.13	3.31	.001	1.53	1.19-1.97
Female sex	0.46	0.05	9.30	<.001	1.58	1.44-1.75
SI history	0.18	0.07	2.69	.010	1.20	1.05-1.37
SA history	0.49	0.15	3.35	<.001	1.63	1.22-2.17
NSSI history	0.30	0.08	3.69	<.001	1.35	1.15-1.59

Note: TGD+Low-Stress and TGD+High-Stress are compared to reference group of non-TGD. SI includes any passive or active suicidal ideation. NSSI = nonsuicidal self-injury; OR = odds ratio; Regr. coeff. = regression coefficient; SA = suicide attempt; SI = suicidal ideation; TGD = transgender and gender diverse; TGD+High-Stress = youth positively endorsing transgender item with high stress; TGD+Low-Stress = youth positively endorsing transgender item with low stress.

**Table 4:** Risk for Suicide Attempt Over Time

	SA (N = 11, 579)					
	Regr. coeff.	SD	*t*	*P*	OR	95% CI
Intercept	–10.31	1.43	–7.21	<.001	0.00	0.00-0.00
Risk factors						
TGD+High-Stress	1.76	0.53	3.33	.001	5.84	2.07-16.47
TGD+Low-Stress	0.88	0.36	2.48	.013	2.42	1.20-4.87
Externalizing	0.03	0.01	2.43	.015	1.03	1.01-1.05
Family conflict	0.09	0.05	1.86	.063	1.09	1.00-1.20
Internalizing	0.01	0.01	1.03	.301	1.01	0.99-1.03
Protective factors						
Acceptance	–0.34	0.29	–1.17	.241	0.71	0.40-1.26
Involvement	–0.04	0.04	–1.06	.290	0.96	0.89-1.04
Covariates						
Age	0.02	0.08	0.28	.777	1.02	0.87-1.20
Deprivation	1.08	0.48	2.26	.024	2.93	1.15-7.45
Female sex	0.45	0.20	2.27	.023	1.57	1.06-2.31
SI history	1.00	0.23	4.44	<.001	2.73	1.75-4.26
SA history	0.62	0.46	1.35	.176	1.85	0.76-4.53
NSSI history	0.66	0.27	2.46	.014	1.94	1.14-3.28

Note: TGD+Low-Stress and TGD+High-Stress are compared to reference group of non-TGD. SA includes interrupted or aborted SAs. NSSI = non-suicidal self-injury; OR = odds ratio; Regr. coeff. = regression coefficient; SA = suicide attempt; SI = suicidal ideation; TGD = transgender and gender diverse; TGD+High-Stress = youth positively endorsing transgender item with high stress; TGD+Low-Stress = youth positively endorsing transgender item with low stress.

#### NSSI

A slight increased odds for endorsing NSSI at least once was observed with increasing age for the overall sample (OR = 1.12, *p* = .001). Compared to non-TGD youth, TGD+High-Stress (OR = 4.81, *p* < .001) and TGD+Low-Stress youth (OR = 2.29, *p* < .001) both experienced significantly greater odds of endorsing NSSI at least once across the study period (Table 3). Among risk factors, higher family conflict (OR = 1.05, *p* = .001), internalizing problems (OR = 1.02, *p* < .001), and externalizing problems (OR = 1.01, *p* < .001) were associated with greater odds of NSSI over the study period. Among protective factors, higher school involvement was associated with significantly lower odds of NSSI over the study period (OR = 0.95, *p* < .001). Among covariates, female sex (OR = 1.58, *p* < .001) and prior history of NSSI (OR = 1.94, *p* < .001) were associated with greater odds of NSSI over the study period.

#### SI

An increased odds for endorsing SI at least once during the study period was observed with increasing age for the overall sample during follow-up (OR = 1.22, *p* = .001). Compared to non-TGD youth, TGD+High-Stress (OR = 4.26, *p* < .001) and TGD+Low-Stress youth (OR = 2.18, *p* < .001) experienced significantly greater odds of endorsing SI at least once across the study period (Table 4). Among risk factors, higher family conflict (OR = 1.07, *p* < .001), internalizing problems (OR = 1.02, *p* < .001), and externalizing problems (OR = 1.02, *p* < .001) were all associated with greater odds of SI over the study period. Among protective factors, both greater parental acceptance (OR = 0.79, *p* = .005) and greater school involvement (OR = 0.94, *p* < .001) were associated with lower odds for SI over the study period. Among covariates, female sex (OR = 1.56, *p* < .001), prior history of SA (OR = 1.64, *p* = .001) and NSSI (OR = 1.41, *p* < .001), greater material deprivation (OR = 1.55, *p* = .001), and older age (OR = 1.21, *p* < .001) were significantly associated with greater odds of SI over the study period.

#### SA

No significant change in risk for SA was observed with increasing age during follow-up. Compared to non-TGD youth, TGD+High-Stress youth (OR = 5.82, *p* < .001) but not TGD+Low-Stress (OR = 2.42, *p* = .013) experienced significantly greater odds of endorsing SA at least once across the study period. No other risk or protective factors were significantly associated with greater odds of SA. Among the covariates, only baseline history of SI was associated with greater odds of SA over the study period (OR = 2.75, *p* < .001).

## Discussion

The present study is the first to examine risk and protective factors for SITB outcomes spanning late childhood through mid-adolescence while accounting for the level of gender-related social stress that TGD youth experienced at home and school. Group comparisons support that TGD youth experiencing higher levels of gender-related social stress (TGD+High-Stress) had more SITB experiences on average compared to TGD youth experiencing lower stress (TGD+Low-Stress) and non-TGD youth, respectively. Results from longitudinal analyses support our hypotheses that TGD youth experiencing high levels of gender-related social stress and TGD youth experiencing low levels of gender-related social stress were at greater risk than non-TGD youth for experiencing NSSI and SI. However, TGD youth experiencing higher but not lower levels of gender-related social stress were at greater risk than non-TGD youth for SA. In addition, we found support for our hypotheses that most known risk and protective factors were significantly associated with NSSI and SI risk even while accounting for gender-related social stress levels.

These results add to prior studies demonstrating disproportionately higher SITB rates among TGD compared to cisgender youth.^5^^,^^6^^,^^10^^,^^45^^,^^46^ In this study, the most robust risk factor for experiencing each SITB outcome belonged to the TGD group with higher levels of gender-related social stress compared to non-TGD youth. This finding is especially important, given that it consistently emerged as the strongest risk factor for all SITB outcomes while accounting for other known robust risk factors (eg, baseline history of SI, NSSI, and SA). These findings support the urgent need for research examining ways to mitigate gender-related social stress for TGD youth. Such research might include identifying pathways to promote TGD-inclusive policies, designing specialized suicide prevention interventions that reflect needs and preferences of TGD youth, and creating safe and supportive communities for TGD youth.^12^

Our results further support that family conflict^20^, ^21^, ^22^ and low levels of school involvement^19^^,^^23^ are risk factors for SITB. Small protective effects were observed for baseline parental acceptance and school involvement for NSSI and SI risk while accounting for gender-related social stress. Positive interactions with caregivers, lower family conflict, higher school engagement, and more community organizations that support school safety through prevention of bullying and harassment are generally protective against SITB for youth broadly and TGD youth.^19^^,^^21^^,^^23^ Having other supportive adults in the community outside of caregivers is also protective against SA in TGD youth with NSSI—a group at high risk for suicide and suicidal behavior.^19^^,^^25^ These risk and protective factors could be potential targets for future SITB preventive interventions among youth broadly and TGD youth specifically.

At present, tailored suicide preventions are lacking for TGD youth, a marginalized and at-risk population. An important next step in preventing SITB in TGD youth might consist of contextualizing what TGD youth find supportive when coping with gender-related social stress. Moreover, TGD youth are a heterogeneous group, with risk and resiliency factors that likely vary across gender identities, culture, race, sex, sexuality, and the interaction among multiple facets of identity.^47^^,^^48^ To our knowledge, no study has examined how multiple aspects of identity support resiliency during identity development—a key developmental milestone of adolescence—for TGD youth. Understanding how identities correlate with risk and resiliency among a diverse range of TGD youth could advance suicide preventions. Until such research is available, suicide preventions for TGD youth might target promoting positive relationships with school peers, families, and appropriate adults in the community.

The present study identified risk and protective correlates of SITB trajectories among TGD and non-TGD youth using data from the largest prospective study of youth in the United States. We were limited to information collected in the original study. Youth were not asked to self-report on gender identities, gender identity expression, and gender contentedness at baseline and at every assessment. There are also important considerations for how research defines gender diverse youth and transgender youth using varied methodologies (eg, self-report with different questionnaires, medical records, etc). Gender incongruence is more commonly self-reported by high school–aged youth in the United States (9.2%), compared to youth who self-report specifically identify as “transgender” (1.8%), and is more common than in youth who receive a medical diagnosis of gender dysphoria (0.9%).^49^^,^^50^ There are also known developmental, sociocultural, and language considerations for measuring gender identity in children,^51^^,^^52^ thus, TGD youth may be underrepresented in this sample. NSSI frequency, severity, and method, and dates for all self-injury are not available. The prevalence of youth self-reporting identifying as TGD (transgender, and/or gender diverse) in the present study (4%) was lower than other US estimates from high school–aged samples of youth who identified as gender diverse and lower than estimates of youth identifying specifically as transgender.^49^^,^^50^ Although it is unclear at present, age ranges for the different samples might play a role in these different prevalence rates.

Results from the present study add to growing concerns about SITB risk among TGD youth. Although higher risk for SITB was observed in TGD youth in the present study, not all TGD youth in general, or even in the present study, experience SITB. Future directions might include applying a more person-centered approach to identify correlates of risk and resiliency within the transgender community specifically. Clinical implications from this study support investing in research that examines ways to bolster coping strategies for gender-related social stress among TGD youth, and facilitating TGD youths’ positive interactions within families and schools to reduce suicide risk. Given that an increasing number of anti-transgender US policies are associated with worse mental health and increased risk for SA among TGD youth,^11^^,^^12^ it is crucial that research identify modifiable risk and protective factors that can be targeted to protect against SITB and suicide in this population.

## CRediT authorship contribution statement

**Amanda J. Thompson:** Writing – review & editing, Writing – original draft, Validation, Supervision, Project administration, Methodology, Investigation, Formal analysis, Data curation, Conceptualization. **Avery N. Abel:** Writing – review & editing, Writing – original draft, Methodology, Investigation, Conceptualization. **Rui Huang:** Validation, Methodology, Formal analysis, Data curation. **Katherine Sarkisian:** Writing – review & editing, Writing – original draft, Methodology, Investigation, Conceptualization. **Mindy Westlund Schreiner:** Writing – review & editing, Writing – original draft, Methodology, Investigation, Conceptualization. **Franky Rife:** Writing – review & editing, Methodology, Investigation, Conceptualization. **Donna A. Ruch:** Writing – review & editing, Supervision, Resources, Project administration, Methodology, Funding acquisition, Data curation, Conceptualization. **Jeffrey A. Bridge:** Writing – review & editing, Supervision, Project administration, Funding acquisition, Conceptualization.
