# Supplementary material for: Trajectories of Self-Injurious Thoughts and Behavior: Risk and Resiliency Among Cisgender and Gender Diverse Youth
Source: JAACAP Open. 2025 Oct 22;4(1):163–71. doi: 10.1016/j.jaacop.2025.10.006 (PMC12925915; doi:10.1016/j.jaacop.2025.10.006)
Supplement: Supplemental Material [file mmc1.docx]

**Missing data within the Study Sample.**

*Missingness across Groups.* There were significant differences in the mean number of missed KSADS suicidality module assessments between TGD+High-Stress, TGD+Low-Stress, and non-TGD, F=(4, 11580) 4.54, *p*=.01. Post-hoc mean comparisons found that those in the non-TGD group on average missed the most assessments (*M*=.23, SD=.60) compared to TGD+Low-Stress (*M*=.15, SD=.47) and TGD+High-Stress youth (*M*=.13, SD=.34). Given inclusion criteria for the study is such that youth must have completed the “*Are you transgender*” item from the KSADS-PL, this is expected.

*Predictors.* There was a significant association between externalizing problems and total number of missed assessments, *β*=.05, *t*(11852) = 5.39, *p*<.001. There was no significant association between parental acceptance, internalizing problems, or school involvement and total number of missed assessments (all *p*’s >.03).

*Covariates.* There were significant differences in total mean number of missed assessments between sex designated as male versus female, *t*(11863) = -2.62, *p*<.001, such that females had a higher number of missed assessments (*M*=.29, SD=.70) compared to males (*M*=.25, SD=.65). There were significant differences in the number of missed assessments between racial groups, F=(4, 11872) 90.45, *p* <.001. Post-hoc mean comparisons found that White children had missed fewer assessments on average (M=.19, SD=.56) compared to children of any other racial/ethnic group, Hispanic children missed more assessments on average (*M*=.32, SD=.75) compared to White non-Hispanic and Other children (*M*=.27, SD=.67), and Black children missed more assessments on average (*M*=.47, SD=.85) compared to children from any other racial group. There was no significant difference, *t*(11850) = .46, *p*=.27, in the average number of missed assessments between those who entered the study with a baseline lifetime history of any SITB (M=.26, SD=.66) compared to those without a lifetime history of SITB (M=.27, SD=.67). There was a significant association between material deprivation, *β*=.08, *t*(11852) = 9.09, *p*<.001, and total number of missed assessments.

Additionally, while it is extremely rare for suicide death to occur under the age of 15 years, it is possible that youth who died by suicide were not identified, and their data would be missing.

**Additional Information of TGD Groupings**

Below, Table 1 and graphically displayed in Figure 1, we report the average gender-related stress levels experienced at each assessment point within each of the TGD groups. In Table 2 and graphically displayed in Figure 2, we report the percentage of youth within each TGD group who affirmed they were transgender on the KSADS-PL at each assessment point. The TGD+Low-Stress group had lower levels of gender-related stress and tended to affirm they were TGD at younger ages compared to the TGD+High-Stress group which experienced higher levels of gender-related stress and tended to affirm they were TGD at older ages.

| **Table 1.**  *Within Group, Mean of Gender-related Stress across Time* | | | | |  |
| --- | --- | --- | --- | --- | --- |
|  | Baseline | 1-yr-fu | 2-yr-fu | 3-yr-fu | 4-yr-fu |
| TGD+High-Stress (n=111) | 1 | 0.73 | 1.36 | 1.31 | 1.63 |
| TGD+Low-Stress (n=379) | 0 | 0.06 | 0.05 | 0.12 | 0.45 |
| *Note:* Mean Problems (0=Not at all, 1=Some, 2=A lot) | | | | | |

**Figure 1**

*Within Group, Mean Gender-Related Stress across Time*

| **Table 2.**  Within Group, Percentage Affirming Transgender Item across Time | | | | | |
| --- | --- | --- | --- | --- | --- |
|  | Baseline | 1-yr-fu | 2-yr-fu | 3-yr-fu | 4-yr-fu |
| TGD+High-Stress (n=111, 1%) | 0.09 | 0.16 | 0.31 | 0.69 | 0.86 |
| TGD+Low-Stress (n=379, 3%) | 0.19 | 0.27 | 0.26 | 0.42 | 0.72 |

**Figure 2**

*Within Group TGD Affirmation across Time*

**Longitudinal Measures of Self-injurious Thoughts and Behavior (SITB)**

The original ABCD study uses the suicidality module to assess SI, SA, and NSSI. Youth report is available at each annual assessment while primary caregiver report is available at every other annual assessment. While there are known discrepancies in youth and caregiver reported self-harm (including NSSI and/ or SA), we used a combined report of both child and caregiver to determine whether youth experienced the specific SITB of interest. Research demonstrates many youth do not report known lifetime history of self-harm at follow-up assessments or may be hesitant to disclose self-harm, thus, we included both reporters. All repeated measures of each specific type of SITB (SI, SA, and NSSI) follow a similar coding structure described below and described in detail in the online supplement and manuscript of some of our own previous research. (*Removed for blind review*) In the ABCD study, the KSADS suicidality module inquires about current (within the past 2 weeks) SITB and lifetime history. Other modules of the KSADS (e.g., substance misuse) ask about symptoms and behavior since the last interactions with the research team. Thus, only the first positive affirmation of lifetime history of SITB can be inferred as a positive event. Below we describe the coding framework for SI, which follows the same logic as NSSI and SA. Please see table below for all items included within each specific SITB by reporter. Positive endorsements of lifetime history (ever occurred) that were not current (defined as within the past 2 weeks of the assessment) could indicate SI occurred between the previous and current assessment. For example, a child may have experienced SI since they last participated in an assessment, but the SI behavior is no longer current when participating in the follow-up assessment. Lifetime history and current SI cannot be disentangled as a separate event (e.g., more than one SI episode, or SI that resolved then was present again later) for youth positively endorsing both current and lifetime history within a single assessment appointment. Thus, only the first instance of lifetime history and all endorsements of current SI respectively were collapsed into new variables defined as SI that had occurred since the last assessment.

| **ABCD Table** | | **Parent Items:** mh_p_ksads_ss; **Youth Items:** mh_y_ksads_ss | | |
| --- | --- | --- | --- | --- |
| **Parent Item** | **Youth Item** | **Item Label** | **Timing** | **Definition** |
| ksads_23_966_p | ksads_23_966_t | Diagnosis - NopastsuicidalideationorbehaviorPast | past | NA |
| ksads2_23_926_p | ksads2_23_926_t | Diagnosis - NopastsuicidalideationorbehaviorPast | past | NA |
| ksads_23_956_p | ksads_23_956_t | Diagnosis - SelfInjuriousBehaviorwithoutsuicidalintentPast | past | NSSI |
| ksads2_23_916_p | ksads2_23_916_t | Diagnosis - SelfInjuriousBehaviorwithoutsuicidalintentPast | past | NSSI |
| ksads_23_964_p | ksads_23_964_t | Diagnosis - AbortedAttemptPast | past | SA |
| ksads2_23_924_p | ksads2_23_924_t | Diagnosis - AbortedAttemptPast | past | SA |
| ksads_23_963_p | ksads_23_963_t | Diagnosis - InterruptedAttemptPast | past | SA |
| ksads2_23_923_p | ksads2_23_923_t | Diagnosis - InterruptedAttemptPast | past | SA |
| ksads_23_965_p | ksads_23_965_t | Diagnosis - SuicideAttemptPast | past | SA |
| ksads2_23_925_p | ksads2_23_925_t | Diagnosis - SuicideAttemptPast | past | SA |
| ksads_23_822_p | ksads_23_822_t | Symptom - Aborted or interrupted suicide attempts Past | past | SA |
| ksads2_23_771_p | ksads2_23_771_t | Symptom - Aborted or interrupted suicide attempts, Past | past | SA |
| ksads_23_825_p | ksads_23_825_t | Symptom - Expect could die from suicide attempt past | past | SA |
| ksads2_23_774_p | ksads2_23_774_t | Symptom - Expect could die from suicide attempt, past | past | SA |
| ksads_23_823_p | ksads_23_823_t | Symptom - Number of suicide attempts Past | past | SA |
| ksads2_23_772_p | ksads2_23_772_t | Symptom - Number of suicide attempts, Past | past | SA |
| ksads_23_816_p | ksads_23_816_t | Symptom - Self-injury intent to die Past | past | SA |
| ksads2_23_765_p | ksads2_23_765_t | Symptom - Self-injury, intent to die, Past | past | SA |
| ksads_23_817_p | ksads_23_817_t | Symptom - Self-Injury thought could die from behavior Past | past | SA |
| ksads2_23_766_p | ksads2_23_766_t | Symptom - Self-Injury, thought could die from behavior, Past | past | SA |
| ksads_23_150_p | ksads_23_150_t | Symptom - Suicidal Attempt Past | past | SA |
| ksads2_23_141_p | ksads2_23_141_t | Symptom - Suicidal Attempt, Past | past | SA |
| ksads_23_824_p | ksads_23_824_t | Symptom - Suicide attempt method Past | past | SA |
| ksads2_23_773_p | ksads2_23_773_t | Symptom - Suicide attempt, method, Past | past | SA |
| ksads_23_962_p | ksads_23_962_t | Diagnosis - PreparatoryActionstowardimminentSuicidalbehaviorPast | past | SI |
| ksads2_23_922_p | ksads2_23_922_t | Diagnosis - PreparatoryActionstowardimminentSuicidalbehaviorPast | past | SI |
| ksads_23_960_p | ksads_23_960_t | Diagnosis - SuicidalideationActiveintentPast | past | SI |
| ksads2_23_920_p | ksads2_23_920_t | Diagnosis - SuicidalideationActiveintentPast | past | SI |
| ksads_23_959_p | ksads_23_959_t | Diagnosis - SuicidalideationActivemethodPast | past | SI |
| ksads2_23_919_p | ksads2_23_919_t | Diagnosis - SuicidalideationActivemethodPast | past | SI |
| ksads_23_958_p | ksads_23_958_t | Diagnosis - SuicidalideationActivenonspecificPast | past | SI |
| ksads2_23_918_p | ksads2_23_918_t | Diagnosis - SuicidalideationActivenonspecificPast | past | SI |
| ksads_23_961_p | ksads_23_961_t | Diagnosis - SuicidalideationActiveplanPast | past | SI |
| ksads2_23_921_p | ksads2_23_921_t | Diagnosis - SuicidalideationActiveplanPast | past | SI |
| ksads_23_957_p | ksads_23_957_t | Diagnosis - SuicidalideationPassivePast | past | SI |
| ksads2_23_917_p | ksads2_23_917_t | Diagnosis - SuicidalideationPassivePast | past | SI |
| ksads2_23_99_p | ksads2_23_99_t | SuicidalideationActivemethodPast | past | SI |
| ksads_23_821_p | ksads_23_821_t | Symptom - Suicidal behavior made preparations Past | past | SI |
| ksads2_23_770_p | ksads2_23_770_t | Symptom - Suicidal behavior, made preparations, Past | past | SI |
| ksads_23_818_p | ksads_23_818_t | Symptom - Suicidal ideation thought of method Past | past | SI |
| ksads2_23_767_p | ksads2_23_767_t | Symptom - Suicidal ideation thought of method, Past | past | SI |
| ksads_23_819_p | ksads_23_819_t | Symptom - Suicidal ideation intent to act Past | past | SI |
| ksads2_23_768_p | ksads2_23_768_t | Symptom - Suicidal ideation, intent to act, Past | past | SI |
| ksads_23_148_p | ksads_23_148_t | Symptom - Suicidal Ideation Past | past | SI |
| ksads2_23_139_p | ksads2_23_139_t | Symptom - Suicidal Ideation, Past | past | SI |
| ksads_23_820_p | ksads_23_820_t | Symptom - Suicidal ideation specific plan Past | past | SI |
| ksads2_23_769_p | ksads2_23_769_t | Symptom - Suicidal ideation, specific plan, Past | past | SI |
| ksads_23_146_p | ksads_23_146_t | Symptom - Wishes/Better off dead Past | past | SI |
| ksads2_23_137_p | ksads2_23_137_t | Symptom - Wishes/Better off dead, Past | past | SI |
| ksads_23_955_p | ksads_23_955_t | Diagnosis - NosuicidalideationorbehaviorPresent | present | NA |
| ksads2_23_915_p | ksads2_23_915_t | Diagnosis - NosuicidalideationorbehaviorPresent | present | NA |
| ksads_23_945_p | ksads_23_945_t | Diagnosis - SelfInjuriousBehaviorwithoutsuicidalintentPresent | present | NSSI |
| ksads2_23_905_p | ksads2_23_905_t | Diagnosis - SelfInjuriousBehaviorwithoutsuicidalintentPresent | present | NSSI |
| ksads_23_953_p | ksads_23_953_t | Diagnosis - AbortedAttemptPresent | present | SA |
| ksads2_23_913_p | ksads2_23_913_t | Diagnosis - AbortedAttemptPresent | present | SA |
| ksads_23_952_p | ksads_23_952_t | Diagnosis - InterruptedAttemptPresent | present | SA |
| ksads2_23_912_p | ksads2_23_912_t | Diagnosis - InterruptedAttemptPresent | present | SA |
| ksads_23_954_p | ksads_23_954_t | Diagnosis - SuicideAttemptPresent | present | SA |
| ksads2_23_914_p | ksads2_23_914_t | Diagnosis - SuicideAttemptPresent | present | SA |
| ksads_23_813_p | ksads_23_813_t | Symptom - Aborted or interrupted suicide attempts Present | present | SA |
| ksads2_23_762_p | ksads2_23_762_t | Symptom - Aborted or interrupted suicide attempts, Present | present | SA |
| ksads_23_814_p | ksads_23_814_t | Symptom - Method of actual suicide attempt Present | present | SA |
| ksads2_23_763_p | ksads2_23_763_t | Symptom - Method of actual suicide attempt, Present | present | SA |
| ksads_23_807_p | ksads_23_807_t | Symptom - Self-injury intent to die Present | present | SA |
| ksads2_23_756_p | ksads2_23_756_t | Symptom - Self-injury, intent to die, Present | present | SA |
| ksads_23_808_p | ksads_23_808_t | Symptom - Self-Injury thought could die from behavior Present | present | SA |
| ksads2_23_757_p | ksads2_23_757_t | Symptom - Self-Injury, thought could die from behavior, Present | present | SA |
| ksads_23_149_p | ksads_23_149_t | Symptom - Suicidal Attempt Present | present | SA |
| ksads2_23_140_p | ksads2_23_140_t | Symptom - Suicidal Attempt, Present | present | SA |
| ksads_23_815_p | ksads_23_815_t | Symptom - Suicide attempt thought could die Present | present | SA |
| ksads2_23_764_p | ksads2_23_764_t | Symptom - Suicide attempt, thought could die, Present | present | SA |
| ksads_23_951_p | ksads_23_951_t | Diagnosis - PreparatoryActionstowardimminentSuicidalbehaviorPresent | present | SI |
| ksads2_23_911_p | ksads2_23_911_t | Diagnosis - PreparatoryActionstowardimminentSuicidalbehaviorPresent | present | SI |
| ksads_23_949_p | ksads_23_949_t | Diagnosis - SuicidalideationActiveintentPresent | present | SI |
| ksads2_23_909_p | ksads2_23_909_t | Diagnosis - SuicidalideationActiveintentPresent | present | SI |
| ksads_23_948_p | ksads_23_948_t | Diagnosis - SuicidalideationActivemethodPresent | present | SI |
| ksads2_23_908_p | ksads2_23_908_t | Diagnosis - SuicidalideationActivemethodPresent | present | SI |
| ksads_23_947_p | ksads_23_947_t | Diagnosis - SuicidalideationActivenonspecificPresent | present | SI |
| ksads2_23_907_p | ksads2_23_907_t | Diagnosis - SuicidalideationActivenonspecificPresent | present | SI |
| ksads_23_950_p | ksads_23_950_t | Diagnosis - SuicidalideationActiveplanPresent | present | SI |
| ksads2_23_910_p | ksads2_23_910_t | Diagnosis - SuicidalideationActiveplanPresent | present | SI |
| ksads_23_946_p | ksads_23_946_t | Diagnosis - SuicidalideationPassivePresent | present | SI |
| ksads2_23_906_p | ksads2_23_906_t | Diagnosis - SuicidalideationPassivePresent | present | SI |
| ksads_23_812_p | ksads_23_812_t | Symptom - Suicidal behavior made preparations Present | present | SI |
| ksads2_23_761_p | ksads2_23_761_t | Symptom - Suicidal behavior, made preparations, Present | present | SI |
| ksads_23_809_p | ksads_23_809_t | Symptom - Suicidal ideation thought of method Present | present | SI |
| ksads2_23_758_p | ksads2_23_758_t | Symptom - Suicidal ideation thought of method, Present | present | SI |
| ksads_23_810_p | ksads_23_810_t | Symptom - Suicidal ideation intent to act Present | present | SI |
| ksads2_23_759_p | ksads2_23_759_t | Symptom - Suicidal ideation, intent to act, Present | present | SI |
| ksads_23_147_p | ksads_23_147_t | Symptom - Suicidal Ideation Present | present | SI |
| ksads2_23_138_p | ksads2_23_138_t | Symptom - Suicidal Ideation, Present | present | SI |
| ksads_23_811_p | ksads_23_811_t | Symptom - Suicidal ideation specific plan Present | present | SI |
| ksads2_23_760_p | ksads2_23_760_t | Symptom - Suicidal ideation, specific plan, Present | present | SI |
| ksads_23_145_p | ksads_23_145_t | Symptom - Wishes/Better off dead Present | present | SI |
| ksads2_23_136_p | ksads2_23_136_t | Symptom - Wishes/Better off dead, Present | present | SI |

**Excluded items:** The following items were not included in the calculations given suicidal intentions cannot be distinguished.

| ksads_23_144_p | ksads_23_144_t | Symptom - Self injurious behavio Past | past | Self-harm |
| --- | --- | --- | --- | --- |
| ksads2_23_135_p | ksads2_23_135_t | Symptom - Self injurious behavio, Past | past | Self-harm |
| ksads_23_143_p | ksads_23_143_t | Symptom - Self injurious behavior Present | present | Self-harm |
| ksads2_23_134_p | ksads2_23_134_t | Symptom - Self injurious behavior, Present | present | Self-harm |
